# Primary care provider perspectives on screening mammography in older women: A qualitative study

**DOI:** 10.1016/j.pmedr.2021.101380

**Published:** 2021-04-17

**Authors:** Sachiko M. Oshima, Sarah D. Tait, Laura Fish, Rachel A. Greenup, Lars J. Grimm

**Affiliations:** a Duke University School of Medicine, Durham, United States; b Department of Family Medicine and Community Health, Duke University School of Medicine, Durham, United States; c Department of Surgery, Yale University, New Haven, Connecticut, United States; d Department of Radiology, Duke University School of Medicine, Durham, United States

**Keywords:** Breast cancer, Cancer screening, Women’s Health, Primary care, Mammography

## Abstract

**Objective:**

Guidelines informing screening mammography for older women are lacking. This study sought to characterize PCP perspectives on screening mammography for patients aged 75 and older.

**Methods:**

This was an exploratory, qualitative study based on semi-structured, one-on-one interviews with PCPs from six clinics affiliated with a tertiary medical center. Two independent coders analyzed interview transcripts and identified themes, subthemes, and representative quotes using inductive analysis methodology.

**Results:**

Ten providers completed interviews. The majority (90%) of providers reported insufficient evidence to suggest a best practice for screening in this population. Providers relied on shared decision-making with patients, a process facilitated by strong provider-patient relationships. Providers took into consideration factors such as functional status, personal risk of breast cancer, and patient preference. Time constraints disincentivized providers to engage in discussions.

**Conclusions:**

PCPs make decisions about screening mammography for older patients on an individualized basis, taking into account patient overall health status and desire for aggressive intervention. They often rely on shared decision-making given unclear clinical guidelines.

**Practice implications:**

These findings suggest that fostering strong provider-patient relationships, addressing patient knowledge gaps, and compensating providers for time spent on counseling may facilitate cost-efficient and patient-centered utilization of screening mammography.

## Highlights

• PCPs believe evidence for screening mammography in older women is insufficient.
• PCPs make decisions regarding screening mammography on a patient-by-patient basis.
• Factors like patient goals of care and functional status influence decisions.
• A strong patient-provider relationship facilitates shared decision-making.
• Time constraints prevent providers from engaging in shared decision-making.

## 1 Introduction

Breast cancer is the second most common cancer among women in the U.S., with an estimated 268,600 new cases in 2019 alone (Howlader et al., 2019). Notably, age remains the most significant risk factor (Colditz and Rosner, 2000). Despite this, evidence related to breast cancer screening and consistent guidelines informing clinical practices are lacking for older women. The U.S. Preventive Services Task Force (USPSTF) 2016 guidelines state that there is insufficient evidence to support mammography recommendations for women ≥75 years (Siu and Force, 2016). In contrast, the American College of Radiology (ACR) recommendations from 2017 suggest starting annual mammography at age 40 and continuing while life expectancy is 5–7 years, while the American Cancer Society (ACS) 2015 guidelines recommend continuing screening while life expectancy is ≥10 years (Smith et al., 2019, Hall, 2018, Monticciolo et al., 2017, Oeffinger et al., 2015). Numerous other organizations release additional conflicting guidelines, making a consensus on best practices unclear (Walter and Schonberg, 2014).

This ambiguity leaves primary care providers (PCPs) with much uncertainty when it comes to recommending screening mammography for older patients. This is reflected in the wide variations in clinical practice in terms of patient counseling and mammography utilization, with high rates of mammography utilization among older women despite conflicting guidelines (Haas et al., 2017, Tan et al., 2014, Onega et al., 2017, Schonberg et al., 2013, Schonberg et al., 2006, Bhosle et al., 2007, Martires et al., 2014, Radhakrishnan et al., 2018, Kotwal et al., 2019). In a 2018 population-based survey of North Carolina residents, 79.8% of women ages 50–74 reported having a mammogram in the last two years, while the rate was similar at 71.8% for women ages ≥75. Of women ages ≥75, 55.2% said their last mammogram was within the last year (Behavioral Risk Factor Surveillance System, 2021). Importantly, breast cancer screening has cost implications both for the patient and the healthcare system as a whole, with an estimated $1.08 billion spent per year in the U.S. Medicare population (Gross et al., 2013). Beyond cost, screening mammography may lead to higher rates of overdiagnosis, or diagnosis of tumors that may not otherwise have been life-limiting. (van Ravesteyn et al., 2015, Etzioni et al., 2013) While prior studies have described factors that might be considered when discussing screening mammography in women aged ≥75 (Walter and Schonberg, 2014, Schonberg et al., 2013, Schonberg et al., 2006, Schonberg et al., 2007, Schonberg et al., 2008, Schonberg et al., 2012), more recent data that reflect provider practices following the release of new guidelines are lacking. Furthermore, much of this prior work has focused on provider perspectives from a limited, urban geographic region. Therefore, this study sought to explore perspectives from a cohort of North Carolina PCPs on screening mammography in older women, factors that influence these practices, as well as barriers to and facilitators of patient counselling.

## 2 Methods

### 2.1 Recruitment

Using the Primary Care Research Consortium affiliated with a tertiary academic medical center, six primary care practices within North Carolina were identified as providing care for older women. All practices support racially and socioeconomically diverse patient populations in different counties within North Carolina (Appendix Table A1) (Survey and Census Bureau, 2019). Utilizing snowball sampling, an introductory email was sent to the physician directors at each clinic site who then contacted providers within their respective practices. In total, this included a convenience sample of 50 physician participants. Providers who responded favorably to the email invitation were sent a survey to collect demographic and clinical training information to assess eligibility criteria. Eligibility criteria included physicians and mid-level providers who self-reported participation in the care of women aged ≥75 in the last year. All providers provided electronic informed consent. This study was approved by the institutional review board (Protocol #00085784).

### 2.2 Interviews

Semi-structured one-on-one phone interviews were conducted with providers during the second half of 2018 and the beginning of 2019 by a trained interviewer (L.F.). The interview guide was developed based on a review of the prior literature and discussions with a breast radiologist, breast surgeon, and a group of PCPs (Schonberg et al., 2006, Schonberg et al., 2014). The guide included questions about providers’ screening mammography practice patterns, especially with regards to older women (Appendix B). Providers were specifically asked about experiences related to discussions regarding the discontinuation of screening mammography as well as barriers to those discussions. Interviews lasted 20–30 min. All interviews were audio recorded and transcribed.

### 2.3 Data analysis

Interview transcripts were analyzed by two independent coders (S.O. and S.T.) using inductive analysis methodology (Gale et al., 2013). A structured coding scheme was developed and tested by both coders independently with a single transcript. Each coder then independently incorporated data from each interview into the coding scheme, focusing on the main ideas that emerged from the data. Coding frameworks from each interview were compared, discussed, and resolved through consensus. Conceptual themes, subthemes, and representative quotes were captured to represent the data using a content analysis framework, which allowed for replicable inferences to be made from the data, guided by contextual content within the interviews (White and Marsh, 2006). Multiple meetings with the study team were held to discuss and affirm the analysis methodology and thematic findings. Saturation over the range of thematic issues was reached after 10 interviews (Hennink et al., 2017). Microsoft Excel was used to organize the data (version 16.35, Redmond, WA).

## 3 Results

Ten provider interviews were completed representing six different primary care clinic locations. The majority of providers were female (60%), medical doctors (90%), and trained in Internal Medicine (60%) (Table 1). The mean number of years in practice since completing training was 12 years. Key themes explaining provider perspectives on screening mammography in women ≥75 are summarized and presented in the following sub-sections with representative quotes presented in Table 2.

**Table 1:** Participant Characteristics.

	N (%) or median (range)
Female	6 (60%)
Degree	
MD	9 (90%)
NP	1 (10%)
Years in practice	12 (3–33)
Type of training	
Internal Medicine	6 (60%)
Family Medicine	4 (40%)

**Table 2:** Key themes representing provider perspectives on mammography in women 75+.

Theme	Subtheme	Representative Quote
Routine screening practices	Shared decision making	“[I will say to patients] … the recommended screening age is the following, we don’t really have a lot of guidelines at your age now, what are your thoughts on this?”
Provider considerations for screening	Health status	“82 year old patient with dementia and significant heart disease and a bunch of other things going on… screening mammogram is the least of their worries.”
	Risk of breast cancer	“This is a complicated area and there are a number of factors that come into play and I think that I would want to consider someone’s risks for having breast cancer –whether there’s illness in family, whether that person might have had other malignancies, gyn[ecologic] malignancies, whether their weight puts them at higher risk, whether they’ve had breast pain, whether they’ve had breast problems in the past. So I would try to incorporate some of those factors into a decision whether they should continue to have mammography or not.”
	Cost	“It depends on their insurance. For the most part, routine screening is covered. I have occasionally had patients who have wonky insurance that have high deductibles and they might be having to pay out of pocket for their mammograms or colonoscopies… When I’m talking about possible downstream effects of a positive screen we don’t necessarily go into the costs of things like surgery or chemo… [or] costs of loved ones maybe having to stop work to take care of them.”
	Patient relationship with provider	“For any age, the better I know a patient the better it is because I know if they have declined other screening tests in the past… but if they are the type of person that always wants to come in and get their things done, then I often will bring up [screening].“
Patient considerations for screening	Desire for continued screening	“I think it depends on their own personal experiences and their experiences with their loved ones in health care. There’s certainly the spectrum of those who have seen people go through a lot with cancer and be sick, or see people at the end of life in the ICU and they say 'absolutely, I don't want that for myself.' Those are the folks that are easier to talk with about doing nothing. The others are people who have in general been fairly healthy or they know someone for whom someone ordered a test and detected something magical and avoided disaster. I think those are the harder people to convince.”
	Desire for intervention	“The whole point is trying to detect a breast cancer that you would find early enough that you can intervene on and so I try to talk through the different scenarios; if we did do screening and we found something that’s concerning, would you want to go through with biopsy? If yes, if found to have cancer would you want to go through with surgery? Would you be willing to do chemo and radiation? So I try to take them through the thought process understanding that it’s not just a test but has a lot of repercussions depending on what the findings are.”
	Patient initiation of discussion	“I actually don't even honestly talk about it or offer it unless they bring it up. So if I have a woman, [age] 76, 77, say[ing] 'I'm due for my mammogram' then we'll talk about it but if it's just someone coming in for their follow-up visit or return, then I don't bring it up.”
Barriers to discussing screening	Time	“As people get older, they have more and more problems and they want to talk about all of them and we’re given 20 min. It’s really hard. I really struggle with that.”

### 3.1 Routine screening

The majority of providers (n = 7) followed USPSTF recommendations for screening mammography in their daily practice, beginning discussions about screening mammography at age 40, with an increased emphasis on routine screening every 1–2 years from age 50–75. Providers also utilized the ACR and ACS guidelines. Two providers included annual clinical breast exams as part of their practice while one recommended self-breast exams. While most providers (n = 7) felt there was inadequate evidence to suggest a best practice for screening mammography in women ≥75, one provider felt there was enough evidence to recommend against screening in older women because “the breast cancers that typically are presenting that late in life tend not to be very aggressive or lethal in my mind so having to go through things at 80 … even something as simple as a routine biopsy, might cause more complications and strife than in the younger population.” All providers reported frequently engaging in shared decision-making with older patients on whether to pursue routine screening mammography, however this was particularly important among the providers who felt that current screening guidelines were unclear.

### 3.2 Provider considerations for screening mammography in older women

Providers reported considering three major factors when deciding whether or not to offer screening mammography to women ≥75: health status, risk of breast cancer, and patient-provider relationship. All providers noted that a patient’s current health status affected how they approached screening mammography recommendations and used overall functional status as an initial decision point on whether or not to discuss mammography, with one provider explaining, “I try not to use age as the end-all be-all cut off.” Some providers (n = 5) assessed health status by thinking about a patient’s 10-year life expectancy through consideration of comorbid conditions, specifically significant renal failure, significant heart failure, significant coronary artery disease, metastatic cancer, and neurologic degenerative processes. Despite the frequent use of this framework, several providers (n = 4) noted uncertainty in their ability to accurately estimate patient prognosis and acknowledged that discussions surrounding life expectancy were difficult to engage in given limited appointment times.

In addition to current health status, several providers took into account a patient’s risk of breast cancer (n = 4). Providers noted they were more likely to bring up screening mammography if there was a family or personal history of breast or gynecological cancer, or if a patient had other pertinent risk factors such as obesity.

Finally, strong relationships between providers and patients gave providers more insight into patient preferences surrounding screening, augmented patient trust of provider recommendations, and helped providers more easily personalize discussions and recommendations. For example, two providers mentioned that they were less likely to engage in discussions regarding screening mammography with a patient if a patient had consistently declined to undergo screening in the past.

Notably, multiple providers (n = 5) reported they did not consider financial costs when discussing mammography with patients, due in part to difficulty determining cost given healthcare pricing opacity. However, one provider suggested that screening mammography was an expendable cost in this age group, saying, “If people are struggling financially and looking for things not to do, I certainly put it out there… as optional.” Similarly, institutional quality metrics were not cited as a consideration in decisions on whether or not to recommend screening mammography. Two providers explicitly mentioned that, while institutional quality metrics were influential in their practice to routinely order screening mammograms in women 40–74, these metrics did not apply for patients ≥75, and thus did not factor into decisions for this patient population.

### 3.3 Patient considerations for screening mammography in older women

Providers discussed a number of patient-level factors that influenced the frequency or length at which screening mammography was discussed during routine visits. These included patient-initiation of discussions about screening mammography, desire for continued screening, and willingness to undergo medical interventions.

Multiple providers (n = 5) noted patient characteristics they believed were associated with a relative increase in patient-initiated discussions around screening mammography; specifically, patients with a perceived younger functional versus chronologic age, those with higher levels of education, and those who had personally investigated the benefits of screening. In the context of compressed appointment times, providers noted that they were more likely to have discussions about the nuances of continued screening with patients who initiated those discussions, even if this was not a priority for their provider.

Once screening mammography was discussed, all providers strongly valued a patient’s desire for continued screening as a rationale for continued use. While all providers perceived that most older women were eager to discontinue screening, they also acknowledged that patients’ personal experiences with screening influenced the degree to which they felt invested in mammography. A few (n = 3) providers mentioned that patients’ prior experiences with breast cancer, both personally and in friends and family members, appeared to shape their perceptions of screening.

When attempting to determine the benefit of continued screening mammography for individual patients, the majority of providers (n = 7) emphasized the importance of knowing the extent to which women were invested in the potential sequela of screening (i.e., additional imaging, biopsies). This was especially important when patients and providers had differing perspectives on whether or not to continue screening. One provider noted, “I try talking with [patients] about whether, if we find something, would you want to know? Would you want any interventions, knowing [that] chemo and radiation and survey are very invasive? …If we find something, would you want to know and have interventions [done], versus do you just want to know, versus do you not want to know, in which case we don’t need to get the screening?”

### 3.4 Barriers to discussing screening mammography in older women

Beyond provider- and patient-level factors, providers identified external barriers that limited conversation regarding screening mammography, including patient satisfaction and time. Several providers (n = 4) mentioned that their recommendations were influenced by their knowledge that physician performance evaluations depend in large part on patient satisfaction. One provider specifically mentioned Press Ganey scores, referring to them as “huge distractors,” and that it was “difficult to completely negate their effect” in patient encounters. This led to provider reluctance to recommend an alternative plan if a patient demonstrated a strong preference for continued screening.

Time was the most important external variable identified by all providers as limiting discussions of screening mammography in patients ≥75. Given that older women tend to have an increased burden of health conditions that must be addressed, providers found it challenging to allow time for a detailed discussion about mammography while managing all of the concerns they are tasked with covering at preventative health visits for these patients. One provider noted, “The big thing is having a very detailed conversation with someone that, quite frankly, a lot of times can be very nuanced in a short amount of time. I think time is the biggest factor.”

### 3.5 Aids in discussing screening

Finally, providers were asked to identify supports that that could assist them in having conversations about screening mammography with this patient population. The majority of providers (n = 6) indicated that a patient-facing handout outlining the current guidelines surrounding screening mammography along with considerations for patients when making decisions about screening would be helpful. Two providers suggested electronic medical record notifications alerting providers that a mammogram is due would be helpful. One provider said reminder alerts with updates on guideline recommendations would be useful, while another mentioned a provider-facing screening decision tool could aid in clinical decision-making.

## 4 Discussion and conclusion

### 4.1 Discussion

Uncertainty persists regarding the value of screening mammography in older women. Providers have been tasked with translating unclear clinical recommendations into clear advice for patients. However, little is known about how providers deal with this uncertainty when counseling older patients on screening mammography, despite the vital role these conversations play in limiting excessive health care utilization and facilitating meaningful discussions around individual health priorities. Screening mammography is associated with significant cost, as an estimated $410 million per year is spent on mammography for women ≥66, (Gross et al., 2013) with questionable survival benefit for older patients who tend to have a higher quantity and severity of comorbid conditions (Muss et al., 2009, Satariano and Ragland, 1994, El-Tamer et al., 2007, Piccirillo et al., 2008). Thus, the identification of factors that influence patient-provider communication around screening mammography has the opportunity to impact patterns of healthcare use and spending.

In this exploratory, qualitative study, we found that the lack of clear evidence on screening mammography for older patients led providers to rely less on data-driven decision-making and more on individualized factors, including overall health status, personal risk of breast cancer, preference for continued screening, and investment in subsequent work-up. An established doctor-patient relationship facilitated shared decision-making regarding continued screening. Time constraints were an important limiting factor for these discussions.

Providers indicated that a strong relationship with their patient was an important mediator that allowed them to communicate effectively regarding the risks and benefits of screening mammography. Prior work has highlighted the importance of communication that values patient preference in making complex clinical decisions in scenarios where no clear answer exists (Ha and Longnecker, 2010, DiMatteo, 1998). For screening mammography specifically, our findings support an evolving body of literature that demonstrates the importance of doctor-patient relationships, including a qualitative analysis of 16 physicians and 23 patients by Schonberg et al. that found longstanding doctor-patient relationships facilitated discussions regarding mammography screening in elderly women (Schonberg et al., 2006).

The shared decision-making around cancer screening enabled by strong provider-patient relationships may help to elucidate patient health priorities and may also reduce unnecessary healthcare utilization for patients who decide that ongoing testing does not align with their goals. For example, in a retrospective review of 509 patients seen at an outpatient primary care clinic, Bertakis et al. demonstrated that patient-centered care was associated with a significant reduction in healthcare utilization and costs, including decreased diagnostic testing (Bertakis and Azari, 2011). Thus, building time into each visit for discussion of patient beliefs, desires, and underlying values regarding their health may not only help to longitudinally strengthen provider-patient relationships, but may also expedite conversations regarding cancer screening while reducing utilization of unnecessary or unwanted screening procedures. To aid in these conversations, providers could consider standardizing the questions they ask patients to get at underlying values that will assist in clinical decision-making.

Importantly, though the benefits associated with this patient-centered approach have been well-documented, we found that there may also be unintended negative consequences. In our study, providers noted that patients with high health literacy were more likely to initiate discussions regarding screening mammography, to indicate a strong preference for continued screening, and to subsequently receive continued screening. This finding is not unique to breast cancer screening. In a survey of 823 participants presenting to an urban public hospital, Yin et al. found that patients with lower health literacy were more likely to rely on a doctor to make health decisions and less likely to consider their own personal preferences (Yin et al., 2012). These disparate levels of patient empowerment may contribute to the differences that have been observed in rates of screening mammography utilization based on race and insurance status (Martires et al., 2014, Rikard et al., 2016). Our findings indicate that the implementation of decision tools, such as those developed by Schonberg et al., 2019, Schonberg et al., 2020, Talking to Patients about Breast Cancer Screening, 2020 in clinical practice could be vital in ensuring all patients are able to make informed decisions about their care and thus empower them to participate in the shared decision-making process (Kadivar et al., 2014).

All providers in our study noted time constraints as a limiting factor to having nuanced discussions with patients regarding screening mammography. These time limitations may not only explain the lack of communication between providers and patients about screening that has been reported in prior studies (Kotwal et al., 2019), but may also account for the documented variability in rates of screening mammography utilization (Martires et al., 2014, Linder et al., 2014). Decision fatigue is a term that describes the phenomenon in which providers are more willing to order healthcare services of questionable utility later in the day when appointment time is limited. Decision fatigue has not yet been documented for mammography utilization but has been documented for other areas of healthcare overuse, such as antibiotic prescriptions, and may contribute to overutilization of screening mammography (Martinez et al., 2018).

Finally, some providers felt pressured to acquiesce to patient preference in situations where patient and provider preference differed in order to maintain high patient satisfaction. Prior work has demonstrated that patients can respond negatively when expected healthcare services are withheld. In a retrospective study of patients presenting with upper respiratory infection symptoms, Martinez et al. demonstrated that patients who received antibiotic prescriptions had higher satisfaction scores than patients who did not (Hojat et al., 2011). While prior studies have demonstrated that PCPs who routinely recommend screening mammography have higher patient satisfaction scores (Melzer et al., 2020), little work has been done investigating patient drivers of screening mammography in women ages ≥75. Future research is needed to evaluate and characterize the patient-provider dynamics that influence excessive screening. In addition, our findings suggest that high-value care may require altered communication architecture and be time intensive. The creation of a billing code that accounts for time spent on shared decision-making and counseling regarding screening mammography for older women may incentivize providers to allocate more of their limited time to these discussions.

Our study included several limitations that must be acknowledged. First, our findings were based on a relatively small convenience sample recruited from clinics associated with a single tertiary academic medical center and may be subject to selection bias and thus not generalizable to the greater PCP population. Second, study findings reflect self-reported data from participants and may not accurately reflect their clinical practice, as prior work has demonstrated that PCP verbalized endorsement of shared decision-making does not always correspond to actual clinical practice.^47^ Importantly, selection bias and/or response bias may have contributed to our finding that all interviewed providers frequently engaged in shared decision-making with their patients regarding screening mammography, as prior literature has demonstrated few patients recall engaging in such discussions with their PCP (Kotwal et al., 2019). Finally, the majority of our participants were medical doctors and thus our data may not reflect the views of advanced practice providers. Future work should be directed towards characterizing the perspectives of community-based and advanced practice providers, as well as those of older patients themselves to more fully identify gaps in knowledge or perceptions that serve as barriers to conversations regarding screening mammography.

### 4.2 Conclusion

Overall, we found that much uncertainty remains within the medical community on the benefit of continued breast cancer screening in women aged ≥75. As a result, screening mammography is guided in clinical practice by both patient- and provider-level factors and is often influenced by external factors, including time limitations. Providers often make clinical decisions on a patient-by-patient basis, taking into account factors such as health status, personal risk, and patient preference. These individualized decisions are best made when fostered by an empowered patient and a strong patient-provider relationship that encourages shared decision-making. However, time constraints often limit the extent to which this form of decision-making is realized, and thus continued improvement is needed to ensure high-value, high-quality care.

## 5 Practice implications

These findings suggest several areas for improvement in clinical practice in order to encourage utilization of screening mammography in women aged ≥75 that is both cost efficient and patient centered. First, providers felt more confident about their decisions regarding screening mammography in this patient population when they had a clear sense of a patient’s goals, highlighting the importance of long-term patient-provider relationships in the primary care setting and suggesting that the implementation of standardized questions that assess goals of care at each visit may be beneficial. Second, providers often felt limited in their discussions with patients by disparities in patient health literacy, indicating that the use of both provider- and patient-facing clinical decision tools may be useful to facilitate informed decisions. Finally, all providers were disincentivized to engage in nuanced discussions about screesning mammography by the time constraints imposed by short appointment times, especially in older patients with numerous other health issues. Thus, accounting for time spent on patient counseling regarding screening mammography through the creation of a specific billing code may help to mitigate these time-related barriers.

## CRediT authorship contribution statement

**Sachiko M. Oshima:** Conceptualization, Formal analysis, Investigation, Writing - original draft. **Sarah D. Tait:** Conceptualization, Formal analysis, Investigation, Writing - original draft. **Laura Fish:** Conceptualization, Methodology, Validation, Formal analysis, Investigation, Writing - review & editing. **Rachel A. Greenup:** Conceptualization, Methodology, Writing - review & editing, Funding acquisition. **Lars J. Grimm:** Conceptualization, Methodology, Writing - review & editing, Supervision, Funding acquisition.

## Declaration of Competing Interest

The authors declare that they have no known competing financial interests or personal relationships that could have appeared to influence the work reported in this paper.

## Figures and Tables

**Table A1:** Primary Care Site Patient Demographic Characteristics by County.

	Durham County N = 3 *Median or %*	Orange County N = 1 *Median or %*	Wake County N = 2 *Median or %*
Age 65 Years and Over	13.6%	14.6%	12.0%
Race/Ethnicity			
Non-Hispanic White	43.0%	69.5%	59.6%
Non-Hispanic Black	36.9%	11.8%	21.0%
Non-Hispanic Asian	5.5%	8.1%	7.7%
Hispanic	13.7%	8.6%	10.4%
Other	1.0%	0.7%	0.9%
Education			
High School Graduate or Higher	88.4%	92.7%	93.0%
Bachelor’s Degree or Higher	48.2%	59.7%	52.8%
Median Household Income	$60,958	$71,723	$80,591
Percent Living in Poverty	14.0%	13.4%	8.0%

Estimates based on the U.S. Census Bureau, American Community Survey, 2019.

## Appendix B Physician interview guide

Introduction

The purpose this study is to understand why women age 75 and older get screening mammograms. Professional societies and governmental bodies provide different recommendations for women in this age group, thus the guidelines are a unclear. Women often rely heavily on their physician’s recommendation about what medical tests, including screening mammography, to undergo. We are interested in hearing your thoughts about screening mammograms in older women and how you decide whether to recommend for or against screening and importantly, when you consider stopping.

1. First, I’m interested in hearing your general opinions about screening mammography in women of any age. Do you routinely recommend screening mammograms for your patients? If so, why? If not, why not?
2. Do you routinely recommend screening mammograms in women age 75 and older? Do you also routinely recommend alternative or supplementary screening strategies?
3. As you may know, breast cancer screening recommendations vary between organizations. What is your understanding of the current screening guidelines and how do they inform your recommendations for women 75 years and older?
4. Do you feel that there is sufficient evidence for or against recommending screening mammograms in elderly women? Why or why not?
5. For elderly women, how does your approach to recommending screening mammograms change compared to younger women? What are the primary patient factors that you consider? PROBE: age, health, functional status, life expectancy, cost
6. What are the primary non-patient factors that you consider? PROBE: guidelines, time for visit, patient-doctor relationship
7. Compared to younger patients, do you feel that older women are more likely to follow your recommendation regarding breast cancer screening? Why or why not?
8. How do you typically discuss screening mammograms with women 75 and older? PROBE: woman wants to continue having mammogram, women doesn’t have opinion about getting mammogram.
9. Have you ever recommended to an older woman that she stop screening mammograms even though she wanted to continue screening? How was that experience? How did you, or would you, approach the discussion with a patient who you feel no longer needs screening mammograms?
10. What do you feel are the greatest barriers to discussing with a patient that she no longer needs screening mammograms? PROBE: time, uncomfortable
11. What could make these discussions easier or more comfortable for you and the patient?

That’s all the questions I have today. Thanks so much.
